# Normalizing inconvenience to promote childhood vaccination: a qualitative implementation evaluation of a novel Michigan program

**DOI:** 10.1186/s12913-020-05550-6

**Published:** 2020-07-23

**Authors:** Denise F. Lillvis, Charley Willison, Katia Noyes

**Affiliations:** 1 Department of Family Medicine, Jacobs School of Medicine and Biomedical Sciences, University at Buffalo, State University of New York, 77 Goodell St., Ste 220, Buffalo, NY 14203 USA; 2 Department of Health Care Policy, Harvard Medical School, 180A Longwood Avenue, Boston, MA 02115 USA; 3 Division of Health Services Policy and Practice, School of Public Health and Health Professions, 270C Farber Hall, Buffalo, NY 14214 USA

**Keywords:** Normalization process theory, Implementation, Immunization programs, Community health education, Organization and administration, County government, Government agency

## Abstract

**Background:**

In 2015, Michigan implemented a rule requiring parents to attend an education session at a local health department (LHD) prior to waiving mandatory child vaccinations. This study utilizes Normalization Process Theory (NPT) to assess program implementation, identifying potential threats to fidelity and sustainability.

**Methods:**

We conducted 32 semi-structured interviews with individuals involved in these education programs across 16 LHDs. Participating LHDs were selected from a stratified, representative sample. One interviewer conducted all interviews using a semi-structured interview guide; two authors coded and analyzed the interview transcripts according to the NPT framework (i.e, sense-making, engagement, collective action, and reflexive monitoring).

**Results:**

There was a lack of consensus about who the stakeholders of this new rule and its resulting program were (sense-making). Perhaps as a result, most LHDs did not solicit advice from key stakeholder groups (i.e., schools, health care providers, community stakeholders) in their planning (engagement). While most interviewees identified providing education and information as the goal, some identified the more challenging goal of persuading vaccine hesitant parents to immunize their children. There was also some variation in perception of who held health educators accountable for meeting the goals of the waiver education program (collective action). Formal program evaluation by LHDs was rare, although some held informal staff debriefings. Additionally, sessions that went particularly well or poorly were top-of-mind (reflexive monitoring).

**Conclusions:**

The immunization waiver education program may be at risk of not becoming fully embedded into routine LHD practice, potentially compromising its long-term effectiveness and sustainability. Managers at the local and state level should maintain oversight to ensure that the program is delivered with fidelity. As the program relies on sustaining inconvenience to encourage parents to immunize their children, any shortcuts taken will undermine its success.

## Background

Vaccine hesitancy, where individuals forgo or are reluctant to receive available vaccinations, was named as a major threat to global health by the World Health Organization in 2019 [1]. In the U.S., state-specific policies influence whether parents can act on their vaccine hesitancy: states decide which vaccines are required and whether parents can decline for nonmedical reasons. Lenient state laws and regulations permitting parents to decline are associated with greater rates of vaccine exemptions [2]. In contrast, stricter exemption laws and regulations lead to a reduction in exemptions and vaccine-preventable disease [3, 4]. States have taken different policy approaches to address rising exemption rates, such as California’s elimination of nonmedical exemptions and Washington’s requirement that parents have a conversation with a health care provider prior to forgoing mandatory child vaccinations [5, 6].

Michigan had among the highest kindergarten nonmedical exemption rates and least effective exemption laws of any state [7, 8]. In 2015, Michigan implemented a new rule requiring parents to attend an in-person education session at a local health department (LHD) prior to receiving a formal waiver from vaccination. Prior to this rule, a parent would only have to sign a form at the school in order to decline mandatory immunizations. The mechanism behind Michigan’s rule and resulting educational program is inconvenience, in that the process for declining immunizations is no longer more convenient than taking a child to receive the required immunizations [9]. Michigan’s immunization waiver education program enjoyed initial success in reducing the prevalence of nonmedical waivers. However, waiver rates have been creeping upward after the program’s first year, though not to pre-program levels [10]. A prior study described the state’s implementation role [11]. The purpose of this study is to assess program implementation from the LHD perspective, identify potential barriers to sustainability and long-term-effectiveness, and propose strategies for improvement.

This study utilizes Normalization Process Theory (NPT) to evaluate the implementation process and identify potential barriers to fidelity and sustainability at the LHD level. NPT was chosen because it focuses on the individual and organizational factors associated with successful long-term program performance. NPT has been widely used to examine how interventions become embedded into routine practice in organizations such as LHDs [12–14]. If an intervention fails to become normalized, program fidelity is at risk and positive long-term results may be harder to attain. Program fidelity is the “degree to which an intervention was implemented as it was prescribed in the original protocol or as it was intended by the program developers” [15]. Fidelity is particularly important to the Michigan immunization waiver education program, as maintaining inconvenience requires that LHDs adhere to the aspects that make the program burdensome to its recipients. Understandably, this burden can anger vaccine-hesitant parents, which in turn exacts an emotional toll on waiver education staff [16]. Staff perceptions of a program as unhelpful to clients negatively influences implementation behavior [17]. Staff will use their discretion to try to make policies more meaningful to their clients and to manage the emotional toll of their work [18, 19]. Administrative theory suggests that LHD staff will seek out ways to lessen the burden on parents in order to lessen the burden on themselves, potentially undermining inconvenience, and fidelity, as a consequence.

Given the nature of Michigan’s approach, it is paramount that LHD staff continue administering it properly. This study evaluates Michigan’s novel local-level intervention, utilizing qualitative interviews with a representative sample of LHDs implementing the program. The purpose is to identify challenges to normalizing the program, and the potential inconvenience it represents to vaccine hesitant parents, into everyday practice. The alternative would be to have the inconvenience aspect erode over time in the face of public pressure—pressure that is present in some localities as well as at the state level [11]. The consequence would be a reduction in policy effectiveness.

## Methods

### Study setting, design, and participants

**Fig. 1:**
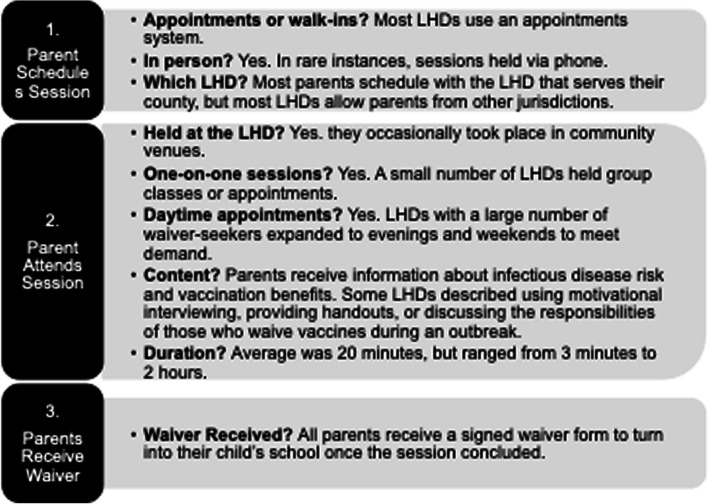
Immunization Education Intervention Description. As a result of a 2015 rule, Michigan parents that want to exempt their child from mandatory school vaccinations for a religious or philosophical reason need to attend an educational session at an LHD and receive a formal waiver form. The intervention process experienced by waiver-seeking parents is described here [11]

This qualitative study utilized interviews from individuals within 16 of Michigan’s 45 LHDs to identify LHD-level implementation barriers to fidelity and sustainability of the state’s immunization waiver education program. Please refer to Fig. 1 for a description of the intervention. For this study, LHDs were selected from a stratified, representative sample developed based on key population characteristics associated with vaccine hesitancy: white race, college education, and higher income levels [20, 21]. We also stratified by population size, as pilot interviews indicated this was important. LHDs were divided into 16 categories based on whether their area served was above or below the state median for these characteristics; no LHDs fit two of the categories. Two LHDs served as interview pilots, bringing the total to 16 LHDs.

Overall, 32 individuals were interviewed from 16 LHDs. Preliminary contact was made with interviewees by email. None of the interviewees were known to the interviewer (DL) prior to this contact, and the other two authors (CW, KN) had no contact with the interviewees. One author conducted semi-structured interviews with between 1 and 4 representatives from each LHD, where interviews averaged 31 min in duration. The two pilot interviews were conducted in person; the remaining interviews were conducted via phone. No non-participants were present during the in-person interviews, and the interviewer was unaware of the presence of any non-participants during the phone interviews. There was a high response rate by LHDs invited to participate (18 LHDs contacted, 16 LHDs consented to interview) and no participants dropped out. The study protocol was reviewed, approved, and determined to be exempt by the University of Michigan Institutional Review Board. Informed consent was obtained from each interviewee prior to participation. During the recruitment and consent process, the identity and affiliation of the interviewer, as well as the topics of interest, were provided to the participants. Interviewees were first screened for involvement with the program. All interviews were conducted between June 2016 and January 2017. Interviews were recorded and transcribed; detailed notes were taken when interviewees declined to be recorded. Please see Table 1 for interviewee characteristics.

**Table 1:** Interviewee Characteristics (*N* = 32)

Characteristic	***N***
Professional Background*	
Nursing or Medical	27
Public Health	7
Business, Law, Public Admin.	5
Implementation Role*	
Managerial	17
Service Delivery	17
Financial	3
Human Resources	3
Other	4
Experience in Field (Years)**	
2 to 5	4
6 to 10	5
11 to 20	8
More than 20	14

*Totals may not add up to 32 because some interviewees fall into more than one category

**One interviewee’s response was unclear and therefore excluded from this table

### Qualitative data analysis

The coding scheme was developed through a directed content analysis [22], which involved an iterative process based on NPT components and interview content. All authors (DL, CW, KN) were involved in assessing whether the components fit our interview data and agreeing upon the interpretation of the components within the study context [23]. NPT consists of four mechanisms, which we adapted as follows [24–26]:

- **Sense-making**: When implementing a new initiative, those responsible for service delivery should have a clear understanding of their role, which includes an identification of the stakeholders that are affected by, and will benefit from, the initiative.
- **Engagement:** Involving stakeholders in the planning of new initiatives assists with embedding by building relationships and fostering ongoing commitment. In particular, their involvement can signal community buy-in to the initiative.
- **Collective action**: Those implementing a new initiative aim to achieve a collective goal determined, in this case, at the state level. However, personally-held goals can either support or undermine the initiative’s original intent. Relatedly, interventions can influence perceptions of responsibility and accountability, which can affect how the tasks are operationalized.
- **Reflexive monitoring**: Once implementation is underway, staff develop perceptions of the new initiative’s usefulness and effectiveness. This perception may be based on formal and/or informal evaluation efforts.

These components are not mutually exclusive. For example, a collective understanding of common goals also provides insights about how those involved in implementation make sense of the program and its benefits. For an illustration of how interview guide questions mapped onto components, please see Table 2. The interview guide developed for this study is provided as Additional File 1.

**Table 2:** Relationship Between NPT Components/Items and Interview Guide Questions

Component or Item	Interview Guide Question*
**Intervention Background**	•It would be helpful for me if you could please describe the education session. Probe: Is it in-person? Is it one-on-one or in a group? Are they held during the day, in the evenings, or on weekends? Did you see any individuals that lived outside your county at your sessions? What materials are provided? How long do the sessions take on average?
**Sense-making**	•When you think of this policy, who are the stakeholders? •[See also Collective Action: What are the goals or objectives of the session?]
**Engagement**	•What role did the following individuals play in developing your department’s educational session? Probe: What feedback or guidance did you solicit from: State officials or state employees Officials or staff within your own health department Officials or staff at another county health department Professional association members Schools or school officials Health care professionals in the community such as physicians or nurses Community members Any other groups or individuals not previously mentioned
**Collective Action**	•What are the goals or objectives of the session? •Who holds you accountable for these goals or objectives?
**Reflexive Monitoring**	•What are the parents’ reactions to the sessions? What issues do parents raise, and how did you respond? Did any change their mind and leave without a waiver? •How did you evaluate the sessions? How did you document success? Probe: Staff member debrief Attendee survey Other feedback from parents

*This is an abridged version of the interview guide. We only included questions or probes relevant to this article. For a full version of the guide, please see Additional File 1

Two authors with qualitative research experience, one a PhD-trained health services post-doctoral fellow (DL) and another a PhD candidate (CW), engaged in line-by-line coding of each transcript. We employed Dedoose software to assist with the organization and coding of themes. We assessed reliability by randomly selecting transcripts from four of the 16 LHDs for a line-by-line coding review. We calculated Cronbach’s alphas for each code across all LHDs: all exceeded a 0.80 threshold. Coding disagreements were discussed and resolved.

To illustrate key themes, we present example quotes in the results section. Additional supporting quotes appear in Additional File 2. To preserve confidentiality, quotes are attributed to individuals with job titles agreed upon by the interviewee. Each LHD was assigned a numeric code that follows the quote in square brackets. This code denotes the Michigan geographic region in which the LHD resides.

## Results

### Sense-making

Across the LHDs interviewed, there was a lack of consensus about who the stakeholders of this new rule and resulting program were. The following were identified as the stakeholders: the community (9 LHDs), parents and/or families (9 LHDs), public health or the health department (6 LHDs), schools (6 LHDs), and local medical providers (4 LHDs). As noted, all were mentioned by less than two-thirds of LHDs interviewed. Additionally, some interviewees had difficulty identifying the rule’s stakeholders. According to an immunization staff member: “It’s difficult to actually identify stakeholders [ …] [Waiver-seeking families] are not partners necessarily. Families seeking the waivers are partners in the process, but they don’t share the same goals. So when you think about traditional stakeholders, not everyone involved in the process shares the same goal” [SE4].

### Engagement

Most LHDs interviewed did not solicit advice from key stakeholder groups in their planning. Less than a quarter indicated that they involved schools, health care practitioners, or members of the community when planning the mandated immunization education sessions. Rather, LHDs were more proactive in informing these groups after the fact. In rare instances (3 LHDs), interviewees discussed involving one or more stakeholder groups in their planning through their involvement in an immunization coalition or child health-focused group. To illustrate, one of the exceptions was a director at a northern health department, which mentioned their LHD’s involvement in a regional group that predated the new waiver education rule: “It’s representatives from health departments, hospitals, private and public schools, pediatricians, and some other key partners in our community that participate. So there was some discussion [of planning the sessions] amongst that group as well” [N1].

### Collective action

While some goals were consistent across LHDs, others deviated in important ways. All LHDs interviewed indicated that providing education and information to the parent is the goal of the rule set forth by the state. An immunization coordinator at a south central LHD stated “Our number one goal is not to change their minds and get them to immunize. Of course, we love that when it does happen, but our main goal is strictly education of the parents of all the requirements and the benefits of vaccinating and the risk of not vaccinating” [SC2]. However, interviewees at 6 LHDs also identified more challenging, and perhaps unattainable, goals that fall outside the original intent of the rule: to persuade waiver-seeking parents to vaccinate their children or to improve local immunization rates. A health officer from an eastern health department said: “The best goal would be to try to answer questions that would allow the parent to feel comfortable getting the vaccine” [E4]. There is some acknowledgement that persuasion is difficult, as exemplified by an immunization supervision staff member from a southeastern health department: “we have our lofty goal of we would love to have them see the light and go and get their immunizations” [SE6].

Related to goals, LHDs differed with regard to their perceptions of who held them accountable for meeting waiver education program goals. The most frequently identified accountability-holders were the state, or specifically the Michigan Department of Health and Human Services, and the LHDs themselves, mentioned by 11 LHDs. Client and public accountability were less-frequently identified, mentioned by 6 and 2 LHDs, respectively. A medical director from a northern health department described accountability as follows: “the school system, and the parents, and the whole public hold the health department to standards of appropriate and professional behavior, in this area and everything else we do” [N1]. And, a manager from a central health department said: “Definitely the Michigan Department of Health and Human Services and my individual local health department because this is a duty that we are charged with, and then the community as a whole” [C4].

### Reflexive monitoring

LHDs seldom engaged in formal evaluations of waiver education program implementation, while informal feedback was much more common. Four LHDs said that waiver-seeking parents may have participated in agency-wide surveys across all their programs. Two LHDs said that they initially conducted a client survey for the waiver program, but they no longer do so. In contrast, 8 LHDs described holding informal staff debriefings specific to the waiver education program. Some, like an immunization program coordinator from a southeastern health department, indicated that they have incorporated discussions of the sessions into their meetings: “We provide feedback to one another about what we’re seeing or what we’re anticipating. If we’re taking appointments and we anticipate somebody maybe more difficult or have specific questions, we’ll offer that information to the staff that may be conducting that appointment. If I see overall themes happening, we talk about that in our meetings” [SE5]. A manager from a central health department explained that their debriefings weren’t regularly scheduled: “generally debriefing is only when there is a situation that we haven’t encountered before, like a parent has a reason that we haven’t really thought of before that we don’t feel like we have enough education on. We come together to make sure that we’re better able to handle that situation in the future” [C4]. Occasionally, LHDs mentioned that, while they do not collect formal feedback, they do receive unsolicited feedback from parents attending the education program.

Interviewees also revealed the parent feedback they received when discussing their experiences delivering the program. Interviewees at 9 of the LHDs indicated negative experiences, as exemplified by a public health nurse from an Upper Peninsula health department: “It’s not a super pleasant experience for me. I just try to meet the parent where they’re at, and offer information that they haven’t really been interested in taking. The people that I’ve seen are very firm in how they feel about it. Just a few parents are verbal and totally object to the sessions” [UP6]. On the other hand, interviewees at 7 of the LHDs relayed positive experiences, such as those described by an immunization staff member from a southeastern health department: “I had one person that came in that were going to get 2 of the 4 vaccines that they needed [ …] then afterwards they got everything. And there was one that came in afterwards and got their child up-to-date and sent our nurse a thank-you card with a box of chocolates” [SE4]. As illustrated by these quotes, sessions that went particularly well or poorly were top-of-mind for interviewees.

## Discussion

Our evaluation identified several threats to the program’s long-term fidelity and sustainability. First, it was rare that LHDs engaged with stakeholders in the community when planning the waiver sessions. Relatedly, there was no clear consensus across the LHDs as to who the stakeholders were in the process. Third, while most identified feasible intervention goals, some indicated that they were aiming at more ambitious, unattainable goals. Fourth, formal evaluation was almost nonexistent, leaving open the opportunity for interviewees to assess the value of the intervention based on anecdotal experiences rather than aggregate results.

This article uses NPT and its components—sense-making, engagement, collective action, and reflexive monitoring—to understand the reasons behind the implementation challenges and examine whether embedding is at risk as a result [24]. Below, we also describe other studies that utilized the NPT framework to identify embedding failures and make suggestions about how to address risks to embedding in program implementation.

Through *sense-making,* implementers develop an understanding of the program to be delivered, which includes their perceptions of the program’s benefits and stakeholders [24]. While common stakeholder groups were mentioned, not all LHDs cited stakeholders such as parents/families and the general community. Additionally, one interviewee revealed a difficulty with the term stakeholder because waiver-seeking parents’ goals conflicted with departmental goals. Difficulty identifying stakeholders indicates that those implementing the program lack clarity about the intervention’s beneficiaries, as well as its value to these groups. This has a cascade effect, as implementers need to know who to engage as a precursor to involving them. Further, without a good understanding of program purpose and value, program staff may focus less on vital aspects of program fidelity. Anecdotal evidence from a Hepatitis B testing study indicated that staff weighed their discomfort of potential racial profiling against the benefit of targeting testing based on nation of birth, choosing not to implement the targeting part of the intervention [14]. Similar to this testing program, conducting the sessions with vaccine hesitant parents can cause staff some emotional discomfort [16]. In contrast, holding in-person educational sessions for vaccine-hesitant parents maintains program fidelity and is necessary to the rule’s mechanism of inconvenience. Immunization program managers at the LHD and state level should emphasize the benefits to children too young to be vaccinated or the immunocompromised in the community, for example, to help offset the discomfort that delivering the sessions presents. Thus, a broader understanding of benefits and stakeholders can assist with program fidelity and lessen negative aspects of program delivery.

*Engagement* addresses the relationship work needed to marshal resources for the program, including stakeholder involvement [24]. Stakeholder engagement provides a unique opportunity for researchers, practitioners, and patients to work together to improve a program’s likelihood of success. This innovative approach allows for a fundamental shift in the research paradigm: from the researcher acting as the sole expert to a partnership in which researchers and stakeholders co-lead research activities. Such an approach allows all stakeholders collectively to utilize their knowledge, skills, and experiences to identify meaningful research questions, develop culturally appropriate study design and data collection strategies, and facilitate dissemination of the research findings [27]. Similarly, human-centered design allows for meaningful involvement of stakeholders to promote sustainability and improvement in health outcomes [28].

Consequently, lack of engagement has consequences for program sustainability. For example, a study of a self-management support program for patients with chronic illnesses found that the intervention was not seen as relevant by either the practitioners or the patients, meaning that neither were willing to invest time and effort in the program [29]. The authors conclude that this is one of several reasons why the intervention failed to become embedded into practice. In this present study, interviews indicated that most LHDs did not engage with external stakeholders when planning the program, missing an opportunity to reinforce the program’s importance among staff and within the community. Engagement may influence program sustainability because it also serves the purpose of building a community coalition. This grassroots work has the potential to offset any backlash against the program by parents burdened by the new waiver process: community-level engagement can create a pool of organized advocates to counter-mobilize, particularly as the rule that created the waiver program is continually under threat in the Michigan state legislature [11].

*Collective action* includes whether the program aligns with the goals and activities of the implementing organization [24]. The vast majority of LHDs hold themselves to the goal of providing education to the waiver-seeking parent. This is also the goal identified in official state documentation about the underlying rule [30]. However, some LHDs also measure their success against generally unattainable goals, notably to persuade vaccine critical parents to vaccinate their child. Persuading vaccine critical parents has been shown to be quite difficult, as those with beliefs on vaccination who are presented with an opposing view generally reject the opposing view [31]. Additional research on waiver education has shown that health educators’ views on the purpose of the sessions evolve over time as they gained experience with the sessions, where persuasion eventually gave way to providing information in a respectful and trusting atmosphere [16]. Yet, this present study, which includes the perspectives of managerial staff in addition to direct service providers, indicates that these views can still persist despite these experiences. Striving for potentially unachievable goals creates challenges to embedding because it is counter to the stated intent of those that hold the LHD accountable, and harms program fidelity as a consequence. Unachievable goals also affect implementer’s perceptions of whether the intervention is working, and therefore worthwhile. Further, such goals indicate competing priorities, which has been shown to lessen the chances of an intervention becoming embedded [32]. Understandably, staff may be grappling with the fact that the mission of a health department is to prevent disease and promote health. Thus, those in management positions at the state and LHD level should continue to emphasize the informational goal of the program, its fit with the department’s mission, and a broad, community-wide view of the program’s stakeholders.

*Reflexive monitoring* includes implementers’ perceptions of the intervention’s impact, which are informed by participant and/or staff feedback and appraisals of the effects of the intervention [24]. With limited exception, LHDs did not formally evaluate the waiver program with parent attendees. Those that did monitor the sessions relied on staff debriefings and informal parent feedback. Additionally, it is not entirely clear whether formally evaluating participant satisfaction in the program would be useful, as the main mechanism behind its success is inconvenience. Yet, other modes of evaluation, such as assessing staff attitudes and knowledge, may reveal items for improvement, such as deviating goals. A prior study of a family violence screening program similarly found that formal evaluation was lacking, which may indicate to staff that the program was not a priority [33]. However, in the present case, it is likely that LHD perspectives are also informed by state-level waiver data, which is highlighted in state health department press releases and receives major media outlet attention. This likely keeps the waiver program on LHD agendas [10, 34].

The key mechanism of the immunization waiver education program, inconvenience, is a relatively new concept in the public administration literature and holds promise for disease prevention and health promotion. Yet, key examples of from the public administration literature focus on the negative consequences of inconvenience, termed an “administrative burden,” in that it can be used to deter qualified individuals from obtaining government assistance [35]. However, inconvenience can be deployed when value of individual rights and autonomy conflict with the pursuit of public health goals—a tension that is often present in the government’s role of protecting the public’s health [36]. One potential application is to limit the sale of tobacco-containing products during certain times of day, such as permitting electronic cigarette purchase only during school hours. This may lessen access for younger students who obtain them from older students. A second potential application is to require stores to locate foods above a certain added sugar threshold a certain distance from the checkout area, thereby rendering them less convenient to purchase. The opioid epidemic represents another application of inconvenience, where databases flag patients that attempt to obtain multiple prescriptions from different doctors and policies limit the number of pills a patient can receive at one time. Yet, in this instance, inconvenience may be causing additional harms that outweigh the benefits. Those with opioid use disorder are now turning to heroin and other drugs that can be purchased on the street. And, pill limits increase the treatment burden on chronically ill patients who must refill prescriptions more frequently. Thus, inconvenience must be wielded carefully, applying only to situations in which burdens are placed on people that can reasonably accommodate them and where the public health benefits outweigh the harms.

As vaccine hesitancy remains a global health concern, countries must consider various approaches to improving vaccination rates. Western democracies prioritize the balance of individual liberties with the protection of the public. Applying inconvenience as a strategy to manage vaccine hesitancy utilizes inconvenience as an incentive that successfully acknowledges individual liberties—while not imposing overly onerous administrative burdens—and encourages individuals to prioritize public health as is necessary for effective vaccine policy implementation. Overall, in the case of vaccine hesitancy, inconvenience is an effective approach that strikes the right balance of respecting individuals’ needs while reducing harm to public health. Future research should address the long-term viability of this inconvenience approach as a method of addressing vaccine hesitancy.

One limitation of this research is that only one interviewee represented the views of 7 of the 16 LHDs. Given the range of responses to certain items, it does not appear that there is an indication of adverse selection. And, in those LHDs where there were multiple interviewees, responses to questions generally aligned across the interviewees. Additionally, our results relied on themes across health departments rather than what is specific to one particular LHD. Finally, this is a cross-sectional analysis of a longitudinal process, meaning that limitations in terms of causality and the inability to capture changes over time apply.

## Conclusion

Using NPT, this evaluation identifies challenges to the implementation of the immunization waiver program that have the potential to undermine fidelity and long-term policy successes. Interviews indicated a limited sense of who the key stakeholders are, which can lead to an inaccurate understanding of (and lack of commitment to) the program. Perhaps as a result, there was a lack of key stakeholder involvement in session planning. This is a lost opportunity to engage allies in favor of vaccination. Although most LHDs indicate that their goals are to provide education, others aim for goals that are more difficult to achieve. While formal evaluation is rare, and may be inappropriate in this case, waiver staff do debrief. However, the experiences shared may emphasize extreme cases, such as those parents who decide to vaccinate or who became angry, which in turn can affect staff perceptions of program value. These program aspects challenge the ability of LHDs to normalize it into regular departmental and immunization clinic operations. At the same time, interviewees are aware that they are accountable to entities such as their LHD and the state. It is imperative that these organizations continue to monitor and guide how LHDs are implementing the program, with the end result being that it remains no less inconvenient to receive the immunizations than it is to opt out.

## Supplementary information

**Additional file 1.** Full Interview Guide. Full IRB-approved interview guide developed for this study

**Additional file 2. ** Additional Supporting Quotes by NPT Component. Examples of the quotes from the study participants illustrating core domains of the Normalization Process Theory

## Data Availability

The de-identified data (i.e., theme-specific quotes) that support the findings of the study presented here are available from the corresponding author upon reasonable request.

## Abbreviations

C: Central Region
E: Eastern Region
LHD: Local Health Department
NPT: Normalization Process Theory
N: Northern Region
SC: South Central Region
SE: Southeastern Region
UP: Upper Peninsula
W: Western Region
