# Supplementary material for: Normalizing inconvenience to promote childhood vaccination: a qualitative implementation evaluation of a novel Michigan program
Source: BMC Health Serv Res. 2020 Jul 23;20:683. doi: 10.1186/s12913-020-05550-6 (PMC7379806; doi:10.1186/s12913-020-05550-6)
Supplement: Supplementary file 1 — Additional file 1. Full Interview Guide. Full IRB-approved interview guide developed for this study [file 12913_2020_5550_MOESM1_ESM.docx]

**Local Health Department Implementation of**

**Michigan Immunization Exemption Law**

**PI: Denise Lillvis**

**Faculty Advisor: Peter Jacobson**

**Interview Guide: Local Health Department Staff**

**Version Date: August 22, 2016**

**Respondent ID: _________________________**

Thank you very much for speaking with me today. [*if consented to record: I have just turned the recorder on*]. I am interested in learning about how you implemented the Michigan school waiver law (or exemption law) that took effect in January 2015. I will be asking you questions about what you did initially for 2015, as well as your plans for the 2016-2017 school year.

1. To start, please tell me a little bit about yourself. What is your professional and educational background? What is your current role in the health department, and how long have you served in this role? [ORGANIZATIONAL FACTORS]
2. In a couple of words, what is your role in implementing the exemption law? Is it managerial, financial, human resources, service delivery, other? [*Ask if not answered in #1 above.*] [SCREENING]

**PROCESS**

I’d like to turn to the process of developing the educational session.

1. Please tell me about the process you used to implement the law. How did you develop the educational session? *Probe:*
   1. *How did you decide what to talk about?*
   2. *What training or materials did you provide to the educators?*
   3. *Where did you get your educational materials?*
   4. *Did you adapt other materials and processes to fit this law, or did you have to start from scratch?* [PLANNING]
2. How much was the state involved in your session planning? Did the state provided written guidance or other policy statements that might indicate how much discretion you were being given to implement the policy? [DISCRETION/INSTITUTIONAL FACTORS]
3. What role did the following individuals play in developing your department’s educational session? *Probe: What feedback or guidance did you solicit from:*
   1. *State officials or state employees (if not addressed above)*
   2. *Officials or staff within your own health department*
   3. *Officials or staff at another county health department*
   4. *Professional association members*
   5. *Schools or school officials*
   6. *Health care professionals in the community such as physicians or nurses?*
   7. *Community members* [ENGAGEMENT]
4. Is childhood vaccination a charged or hot button issue in the area served by your LHD? Why or why not? In what ways did this affect how you approached the sessions? [POLITICAL FACTORS] *Probe: What is the role of vaccine-critical groups in your community? In what ways has their presence affected implementation of this rule?*
5. What were your top considerations when designing the education sessions? [O/ I/ P FACTORS]
   1. *Resources (Staffing/Financing)*
   2. *Constituent needs*
   3. *Competing priorities*
   4. *Other organizational factors or trends*
   5. *Other external factors or trends*

**STRUCTURE/CHARACTERISTICS**

Now, I’d like to talk a bit about the educational sessions themselves.

1. It would be helpful for me if you could please describe the education session. *Probe:*
   1. *Is it in-person?*
   2. *Is it one-on-one or in a group?*
   3. *Are parents required to bring their children?*
   4. *Are they held during the day, in the evenings, or on weekends?*
   5. *Did you see any individuals that lived outside your county at your sessions?*
   6. *What materials are provided?*
   7. *Is it primarily a presentation, more of a Q/A, or a discussion with parents?*
   8. *How long do the sessions take on average?*
   9. *How many education sessions have you delivered?*
   10. *When was the first one held?* [VARIATION]
2. What funding is available for the sessions, perhaps from the state or county government? Alternately, how much does it cost to implement the policy? [RESOURCES/ ORGANIZATIONAL FACTORS]
3. How many health department staff members deliver the sessions, and how do you find staff for the education session? How does implementing the law affect your workflow? [RESOURCES/ ORGANIZATIONAL FACTORS]
4. How do parents find out about the required session? What was the health department’s role in raising awareness about the requirement? [COMMUNICATIONS]
5. What are the goals or objectives of the session [GOALS*]
6. Who holds you accountable for these goals or objectives? When you think of this policy, who are the stakeholders? *Potential responses: DHHS, Parents, Lawmakers, the General Public* [POLITICAL FACTORS]
7. Tell me about the parents and guardians who attend the education session. What are the demographics of the attendees? *Probe:*
   1. *What are the main reasons individuals want to exempt their children?*
   2. *Political beliefs?*
   3. *Basis of their vaccine hesitancy?*
   4. *Demographic characteristics?Did any of the attendees surprise you?* [VARIATION/POLITICAL FACTORS]

**OUTCOMES/EVALUATION**

I’m now going to ask some questions about the results of the sessions, how you evaluate the sessions, and anticipated changes in the sessions for the 2016-2017 school year.

1. What are the parents’ reactions to the sessions? What types of questions to they have? What issues do parents raise, and how did you respond? Did any change their mind and leave without a waiver? [ATTITUDES]
2. How did you evaluate the sessions? How did you document success? *Probe*:
   1. *Staff member debrief*
   2. *Attendee surveyOther feedback from parents?* [EVALUATION]
3. What data about the sessions do you report to the state? What data about the sessions do you collect or report internally? [EVALUATION]
4. What changes are you making to the education sessions this year, and what are the reasons for the changes? *Probe:*
   1. *In what ways did implementing the law work well in 2015?*
   2. *What will you definitely repeat in your sessions this upcoming year?*
   3. *In what ways did the educational program not work as well?*
   4. *What will you definitely not repeat in your sessions this upcoming year?* [O/I/P FACTORS]
5. Why do you think the waiver rate in your area increased/decreased/stayed the same? *Probe: does it have to do with:*
   1. *Characteristics about area that you serve? [If LHD incorporates multiple counties, ask about this]*
   2. *Characteristics about the people/parents that you serve?*
   3. *Funding, staffing, or other issues internal to your LHD*
   4. *Issues coordinating or receiving guidance from the state?* [VARIATION, O/I/P FACTORS]
6. Are you aware of any other programs or efforts that may have affected the exemption rates in your county? *Probe:*
   1. *Public awareness campaign about the importance of vaccination*
   2. *Increased access to vaccination at the health department*
   3. *Increased enforcement efforts to make sure children were vaccinated or had waivers* [CONFOUNDERS]

**CONCLUDING QUESTIONS/REQUESTS**

1. Is there anything you would like to add to our conversation today?
2. Would you be willing to share any documents related to the education sessions, such as a PowerPoint or other materials you provide to parents?
3. [*If not already addressed*] Who else would you recommend that I speak to about implementing this new law?
