# Supplementary material for: Normalizing inconvenience to promote childhood vaccination: a qualitative implementation evaluation of a novel Michigan program
Source: BMC Health Serv Res. 2020 Jul 23;20:683. doi: 10.1186/s12913-020-05550-6 (PMC7379806; doi:10.1186/s12913-020-05550-6)
Supplement: Supplementary file 2 — Additional file 2. Additional Supporting Quotes by NPT Component. Examples of the quotes from the study participants illustrating core domains of the Normalization Process Theory [file 12913_2020_5550_MOESM2_ESM.docx]

**Appendix 1. Additional Supporting Quotes by NPT Component**

| **Component** | **Interviewee Quote** |
| --- | --- |
| **Sense-making** | **A health officer from an eastern health department:** “I always go back to stakeholders primarily being our local population of our county. I think that with immunization, you really have to look beyond just the children who have waivers. […] Unimmunized children are a threat to the rest of the children in school and in the community, and frankly a threat to the rest of the population as a whole” [E4]. |
| **Engagement** | **A director from a central health department:** “We didn't talk to any of those entities about how we designed it. But, we did send information out to all the schools in our counties that basically made sure that they were aware of the change” [C3].  **A nurse from an eastern health department:** “We did speak with our local providers that maybe had signed medical waivers, so that they knew that they could still continue to sign medical waivers” [E2]. |
| **Collective Action** | **A manager at a central health department:** “the goal is to provide the most accurate and reputable information on the benefits of vaccinations, associated side effects, and the risks associated with waiving vaccines to the child and the community at large” [C4].  **A public health nurse from a western health department:** “[the goal is] to make sure that the parents have an understanding of why they're choosing [not] to vaccinate outside of because ‘I read this on the internet,’ just to make sure that they know that there's evidence-based research for these vaccines and the safety, and that they are making an informed decision” [W5]. |
| **Reflexive Monitoring** | **An immunization coordinator from a northern health department:** “We get on-the-spot, anecdotal feedback that we've responded to” [N1].  **A director of the public health division of an Upper Peninsula health department:** “Parents can always send issues by e-mail or they can call some of the higher-ups here at the health department if they have complaints, or issues, or questions, or anything like that” [UP1]. |
